# Prevalence and Perceptions of Illicit Substance Use Amongst Medical Students

**DOI:** 10.15694/mep.2021.000163.1

**Published:** 2021-06-08

**Authors:** Matthew Palin, Kevin McConville

**Affiliations:** 1 University of Dundee

**Keywords:** Medical students, illicit drugs, professionalism

## Abstract

This article was migrated. The article was marked as recommended.

**Background:** The General Medical Council state illicit substance use by medical students is an example of unprofessional behaviour. Previous research has shown the use of illicit substances to exist amongst medical students in the United Kingdom. This research aimed to determine the prevalence of illicit substance use amongst a single cohort of medical students and gather information concerning perceptions of this behaviour. The study sought to quantify the prevalence of illicit substance use within each year of study and gender identity.

**Methods:** An anonymous online questionnaire was employed to conduct this quantitative research. This included nine questions regarding illicit substance use amongst medical students, with three additional demographic questions. The Statistical Product and Service Solutions was utilised to interrogate the data.

**Results:** Out of 927 students in the medical school 201 (21.7%) people completed the questionnaire. 50.7% of respondents reported an any lifetime use of illicit substances, with 20.9% of the cohort reflecting recent illicit substance use within the previous 30 days. Drug use included amphetamines, amyl nitrate, cocaine and ecstasy amongst others. Cannabis was the most commonly used illicit substance, with a lifetime use of 45.8%. Statistical significance was determined for use of illicit use of substances within the male gender and older cohort years of the medical school.

**Conclusions:** The current cohort had a greater prevalence of cannabis use than previously determined amongst medical students. Males and students in older years had higher rates of illicit substance use than their respective demographics. As such, further research may be necessary to investigate the underlying reasons for these findings.

## Introduction

The process of leaving secondary school and becoming a university student may provide an opportunity for one to experience true liberty and be free from parental control. However, it has been suggested that this freedom may result in students’ experiencing unsafe accommodation, difficulties with studies and issues with loved ones ( Sommet *et al.*, 2012). As such, this time can be heavily linked with trialling of illicit substances and can be when this behaviour commonly increases ( Locke *et al.*, 2015).

A recent study investigated the phenomenon of illicit substance use by British university and college students. The National Union of Students (NUS) surveyed 2810 students at 151 higher and further educational institutions entitled ‘Taking the Hit’ ( NUS, 2018). They observed 56% of respondents had used illicit substances at some point, with 39% reporting current use ( NUS, 2018), perhaps suggesting a behaviour seen regularly within student culture.

For the purposes of this research, we have defined illicit substance use as:

“The non-medical use of a variety of drugs that are prohibited by international law”

( Degenhardt, 2004)

Given the high level of illicit substance use amongst university students on a global scale ( Sommet *et al.*, 2012; Suerken *et al.*, 2014) it might be viewed as essential to consider the impact this will have specifically within the medical student population. This General Medical Council (GMC) addresses illicit substance use in their guidance for medical students ( GMC, 2016, 2018).

One such example, ‘Achieving Good Medical Practice’ ( GMC, 2016) reminds students that they will become part of a “trusted profession” and are required to demonstrate professional behaviour throughout their time at medical school. This guidance goes on to state that one such example of unprofessional conduct is substance misuse ( GMC, 2016). Furthermore, according to the most recent Outcomes for Graduates ( GMC, 2018), newly qualified doctors have the professional responsibility to prevent any patient risk which may be caused by their behaviour, an example being proscribed substance abuse. Thus, the main aim of this study was to investigate the prevalence of illicit substance use and its perceptions amongst medical students in one institute.

## Methods

*Research Design:* This study made use of survey research methodology through an anonymous, online questionnaire ( Joint Information Systems Committee, 2020) (Supplementary File 1). The questionnaire was sent out to all students at a single medical school in the United Kingdom (UK). This questionnaire was subsequently analysed quantitatively. It was hoped that this research would give a better insight into illicit substance use amongst this group and lay the groundwork for further research into this subject.

This method was felt most appropriate by the researchers as it allowed for data to be easily gathered ( Ponto, 2015). Moreover, by using an online questionnaire, anonymity was provided to the participants in this study ( Lavrakas, 2008). This was felt necessary as participants in the survey may disclose illegal behaviour. As such, participant identity would not be revealed to the researchers nor higher authorities.

*Data Collection:* At the selected medical school, the degree consists of a five-year programme. All students in Years 1-5 were invited, via an intermediatory ‘gate-keeper’ to take part during the period 6/2/2020 - 29/2/2020. Furthermore, students who have completed their Year 3 studies have the option to intercalate and study a Bachelor of Medical Science (BMSc) degree for one year, resulting in a sixth year group. Given this, there were a total of 927 potential participants.

*Data Analysis:* Statistical analysis was applied using Statistical Product and Service Solutions (SPSS) Version 26.0 (International Business Corporation, 2019). Chi-Squared Tests were utilised to ascertain any statistical significance between the responses and the demographic categories of year of study and gender identity. This form of statistical analysis was believed to be suitable as these tests compare the spread of categorical variables in two different samples ( Swinscow, 1997). P values < 0.05 were considered to be significant. Chi-Squared tests were utilised to determine statistical significance for the relationship between Year of Study/Gender Identity and the questions posed. Specifically, this involved creation of a 2x2 contingency table. Therefore, the options for Year of Study were merged into two groups - Lower School (comprising Years 1, 2 and 3) and Upper School (including Years 4 and 5 and BMSc). There were 2 respondents who selected ‘Other’ or ‘Prefer not to say’ when asked their gender identity. Edwards (1997) proposes that if <5 respondents select a certain demographic, these results are not suitable for Chi-Squared tests. As such, these results were not included in the appropriate analysis.

*Ethical Considerations:* Ethical approval for the undertaking of this study was requested from and granted by the School of Medicine Research Ethics Committee (SoMREC) - SoMREC reference number SMED20/14.

## Results/Analysis

*Population Data:* Out of the 927 students in the medical school 201 (21.7%) completed the questionnaire. Within the survey, each respondent was asked to provide data for two different demographic categories. These were: their current year of study ( Table 1) and gender identity ( Table 2).

**Table 1:** Summary of Current Year of Study

Lower or Upper School	Current Year of Study	N	Total Number of Students in Each Year	Response Rate for Students per Year (%)	Total Number of Students in Lower and Upper School	Response Rate for Students per Upper and Lower School (%)
**Lower School**	First	48 (23.9%)	204	23.5	535	**22.1**
Second	32 (15.9%)	147	21.8
Third	38 (18.9%)	184	20.7
**Upper School**	BMSc	29 (14.4%)	84	34.5	392	**21.2**
Fourth	40 (19.9%)	172	23.3
Fifth	14 (7.0%)	136	10.3
	**201** ( **100.0%**)	**927**	**21.7**	

**Table 2:** Summary of Gender Identity

Gender Identity	n
Female	150 (74.6%)
Male	49 (24.4%)
Other	1 (0.5%)
Prefer not to say	1 (0.5%)

**Figure 1:**
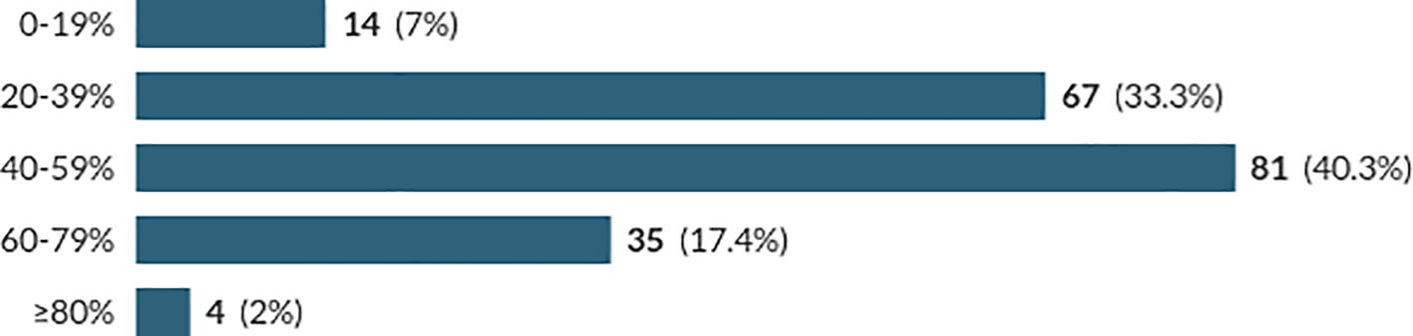
Perceived Percentage of Medical Students who have Ever Used an Illicit Substance

**Figure 2:**
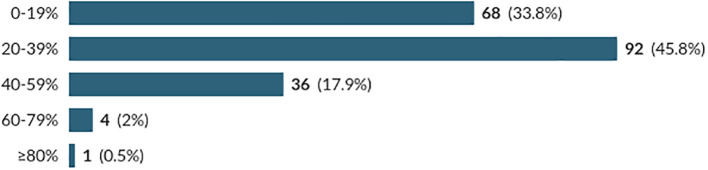
Perceived Percentage of Medical Students who had Recently Used an Illicit Substance

*Perceived Level of Illicit Substance Use:* The first two questions of the survey concerned the respondents’ perceived level of illicit substance use within the medical school cohort. Question 1 regarded the perceived number of medical students who had ever used an illicit substance ( Figure 1) while Question 2 related to the perceived number who had used an illicit substance within the last 30 days ( Figure 2). Students were asked to select the percentage of their student cohort who they believed had either used an illicit substance at any point in their lifetime or had used an illicit substance within the last 30 days.

*Actual Level of Illicit Substance:* Question 3 and 4 concerned the actual level of illicit substance use within the medical school cohort. Question 3 asked the respondent if they had ever used an illicit substance at any point in their life ( Table 3) while Question 4 queried if the respondent had used an illicit substance last 30 days ( Table 4).

**Table 3:** Number of Respondents who have Ever Used an Illicit Substance

Ever Use of an Illicit Substance	N
Yes	102 (50.7%)
No	99 (49.3%)

**Table 4:** Number of Respondents who have Recently Used or Currently Use an Illicit Substance

Current/Recent Use of an Illicit Substance	N
Yes	42 (20.9%)
No	159 (79.1%)

*Specific Illicit Substances Used:* Question 5 concerned the specific substances used by the individual respondent ( Table 5). Each respondent was asked to select any illicit substance they had ever used from the list provided. Furthermore, the option of ‘other’ was given to allow participants to state any illicit substance they had used but was not listed within the question. If a respondent had never used an illicit substance, they were asked to select the option ‘none’.

**Table 5:** Number of Respondents who have Ever Used a Specific Substance

Specific Substance	N
Amphetamines	4 (2%)
Amyl Nitrate	37 (18.4%)
Anabolic Steroids	0 (0.0%)
Benzodiazepines	6 (3%)
Cannabis	92 (45.8%)
Cocaine	37 (18.4%)
Ecstasy/MDMA	45 (22.4%)
GHB	0 (0%)
Ketamine	29 (14.4%)
LSD	9 (4.5%)
Magic Mushrooms	6 (3%)
Nitrous Oxide	28 (13.9%)
Opioids	3 (1.5%)
None	99 (49.3%)
Other	3 (1.5%)

*Time of First Use:* Question 7 concerned the time at which the respondent first used an illicit substance ( Table 6). This was specifically in regard to whether this occurred before or after they had matriculated at the university’s school of medicine.

**Table 6:** Time of First Use of an Illicit Substance

Time of First Use	N
Before Medical School	58 (56.9%)
After Medical School	44 (43.1%)

*Statistical Analysis and Significance:* The answer options for question 1 were merged into two groups: 0-59% and ≥60%. The Chi-Squared Tests show statistical significance between individuals who responded either ‘0-59%’ or ‘≥60%’ to Q1 to being in the lower or upper school (p<0.001). This suggests that participants in the upper school are more likely to believe ≥60% of students have ever used illicit substances.

It was shown to be statistically significant that participants in the upper school were more likely to have ever used an illicit substance (p=0.024). Furthermore, for students who identify with a male gender, it was statistically significant that they were more likely to have both ever used an illicit substance (p=0.002) and have used an illicit substance within the last 30 days (p<0.001).

With regard to ever use of a specific illicit substance, it was shown, with statistical significance, that males were more likely to have ever used cannabis (p=0.005), ecstasy/MDMA (p=0.006), cocaine (p=0.013) and amyl nitrate (p=0.013). Furthermore, it was found that it was statistically significant for participants in the upper school to be more likely to have used ecstasy/MDMA (p=0.027) and cocaine (p=0.004).

Finally, concerning time of first use, it was shown to be statistically significant that participants in the lower school were more likely to have first used an illicit substance before matriculating at medical school. Participants in the upper school were therefore more likely to have first used an illicit substance whilst at medical school (p=0.030).

## Discussion

50.6% of all respondents answered they had ever used an illicit substance. However, when dividing this figure into year of study, it could be broadly observed that the proportion of respondents who have ever used an illicit substance increases as the year of study progresses (first-year 35.4% vs fourth-year 52.5%). This is also noted in previous literature, where it was observed that in terms of ever use of an illicit substance, 35.1% of first-year respondents reported previous use compared with 57.0% of final-year respondents ( Bogowicz *et al.*, 2018). Furthermore, the BMSc cohort had the highest percentage of both ‘ever use’ (72.4%) and ‘recent use’ (35.5%). This may reflect the fact that respondents in this year have completed three years of medical studies alongside the researcher and may feel more comfortable in expressing any illicit substance use. In addition, one could argue the corresponding increase in ‘free-time’ compared with Years 1-3 of the medical degree, when studying a BMSc may provide a higher degree of opportunity for illicit substance use. However, as no previous research has considered illicit substance use amongst an intercalated year group, this may require further analysis.

20.9% of all respondents had used an illicit substance in the 30 days prior to submitting their response. Similarly, within year of study, a greater proportion of students’ report ‘recent use’ the further into the medical programme they progress (first-year 12.5% vs fourth-year 22.5%). This contradicts previous literature, who found a fall in the percentage of medical students who had recently used an illicit substance when comparing fourth-year to first-year ( Bogowicz *et al.*, 2018).

The most common illicit substance that had been used by respondents at any point previously was cannabis (45.8%). This is also noted in similar evidence ( Bogowicz *et al.*, 2018; Newbury-Birch, Walshaw and Kamali, 2001). In terms of year of study, it was found that 35.4% of first-year respondents; 50.0% of second-year respondents; 39.4% of third-year respondents and 50.0% of fourth year respondents had ever used cannabis. This could suggest that the further medical students progress in their studies, they are more likely to have used cannabis (35.4% in first-year vs 50.0% in fourth year). It may be of interest to determine the reasons for this, especially as it may be assumed that older medical students are more likely to display “professional behaviour”.

The illicit substance with the second-highest rate of ‘ever use’ was ecstasy (22.4%). Interestingly, this finding was also noted in international research, as a similar piece of research also observed ecstasy had the second-highest rate of use amongst a cohort of Greek medical students ( Papazisis *et al.*, 2017). Furthermore, research from 20 years previously found that the second most popular used illicit substance amongst UK medical students was amphetamines ( Newbury-Birch, Walshaw and Kamali, 2001). It therefore could be argued that in the previous two decades, the most popular ‘upper’ used by UK medical students has changed from amphetamines to ecstasy.

It was found that participants from Years 1, 2 and 3 were more likely to believe 0-59% of medical students had ever used an illicit substance, while those in BMSc and Years 4 and 5 were more likely to believe that ≥60% of medical students had ever used an illicit substance to a statistically significant degree (p<0.001). This finding could be explained by the increased tenure of students in the upper school, providing a greater opportunity to experience illicit substance use amongst their peers. However, there has been no prior research into medical students’ perceived use of illicit substances by fellow medical students. As such, this finding may require greater research.

Furthermore, it was found to a level of statistical significance that participants in BMSc and Years 4 and 5 were more likely to have ever used an illicit substance (p=0.024). Participants who identified as male were also more likely to have ever used (p=0.002) and have recently used an illicit substance (p<0.001). This corresponds to research in the United States, which observed that male medical students were more likely to have ever used an illicit substance and have recently used cannabis ( Ayala *et al.*, 2017). As such, it may be important to understand the significance of this finding by conducting further qualitative research to comprehend the reasons behind this behaviour amongst those who identify as male.

## Conclusion

Illicit drug use by medical students continues to remain an issue within some medical schools. Drug consumption by intercalating degree medical students is a previously unexplored area. Changing trends might suggest that more senior, male medical students are now, not adverse, to illicit drug use - reasons for this need explored.

## Take Home Messages

• Illicit substance use takes place amongst medical students and this behaviour is recognised
• Males and participants in Years 4 and 5, as well as intercalating degree students, were more likely to have ever used and recently used illicit substances
• Cannabis was the most commonly used illicit substance, both in terms of ever use and recent use
• Participants in Years 1-3 were more likely to believe that a fewer number of students had ever used an illicit substance
• Further qualitative research may be useful in determining the reasons behind illicit substance use amongst medical students

## Notes On Contributors

**Matthew Palin** has obtained his BMSc Med Ed (Hons) at the University of Dundee. Currently he is a fourth-year medical student and is an Associate Fellow of the Higher Education Academy.

**Kevin McConville** studied medicine at the University of Dundee and obtained his EdD at the Open University. Currently he is the acting head for the General Practice undergraduate team within Dundee Medical School, co-programme lead of the BMSc Medical Education degree and Senior Fellow of the Higher Education Academy.
