# Supplementary material for: Prevalence and Perceptions of Illicit Substance Use Amongst Medical Students
Source: MedEdPublish (2016). 2021 Jun 8;10:163. [Version 1] doi: 10.15694/mep.2021.000163.1 (PMC10939573; doi:10.15694/mep.2021.000163.1)
Supplement: Peer review history

# Reviewer report: Reviewer response for version 1

**DOI:** 10.21956/mep.19109.r27559

**Published:** 2021-07-09

**Authors:** Richard Hays

**Affiliations:** 1 James Cook University

This review has been migrated. The reviewer awarded 4 stars out of 5 This article is a timely reminder that medical students are human and that medical educators need to consider drug use when managing academic performance and personal crises in medical students. As with our patients, we need to ask the right questions. A weakness of the research is that the response rate was low, perhaps partially because of the topic, so the incidence data may not be reliable. One minor issue is that in Table 1 the responses should be reported as n (%) to provide a more compete picture. I could not see Figures 1 and 2 - the system was being worked on at the time of the review so may have been hidden somewhere - and those data should have been mentioned in the text. A question that perhaps should have been asked was whether or not the students thought that drug use was a problem in medical careers, but that is probably another study. This article is a worthwhile read for all medical educators and regulators.

Reviewer Expertise:

NA

No decision status is available

# Reviewer report: Reviewer response for version 1

**DOI:** 10.21956/mep.19109.r27558

**Published:** 2021-06-30

**Authors:** Ben Canny

**Affiliations:** 1 Monash University

This review has been migrated. The reviewer awarded 4 stars out of 5 This manuscript represents an important addition to the medical education literature. Importantly, it shows that medical students do not differ significantly from the rest of the publication with respect to illicit drug use. Particular powers of the study include the facts that the survey was conducted anonymously, and one of the respondents' peers was intimately involved with the research.I was surprised that among the demographic data being collected, there was no reference to the age of the respondent. While recognising that in the particular cohort (due to selection issues) this range would most probably have been narrow, some additional important observations may have emerged.It was of interest that use increased with duration during the course. The authors are correct in pointing out that this observation, and some others, warrants further examination, especially given the potential professional implications of illicit drug use for medical practitioners. The observation that use increases as one approaches graduation and professional registration, where the professional consequences of illicit behaviours are arguably greater, might appear counter-intuitive at first, but probably just confirms "normal" behaviour in society. Consideration should be given to the education of all medical students, especially more senior ones, about the likely sanctions for such behaviours.Overall, I commend this paper to the medical education community.

Reviewer Expertise:

NA

No decision status is available
